# Characteristics and outcomes of a cohort hospitalized for pandemic and seasonal influenza in Germany based on nationwide inpatient data

**DOI:** 10.1371/journal.pone.0180920

**Published:** 2017-07-14

**Authors:** Daniel von der Beck, Werner Seeger, Susanne Herold, Andreas Günther, Benjamin Löh

**Affiliations:** 1 Universities of Giessen and Marburg Lung Center (UGMLC), Member of the German Center for Lung Research (DZL), Giessen, Germany; 2 Agaplesion Lung Clinic Waldhof Elgershausen, Greifensstein, Germany; Chang Gung Memorial Hospital, TAIWAN

## Abstract

**Rationale:**

From June of 2009 to August of 2010 the influenza subtype H1N1pdm09 caused a worldwide pandemic. The impact on populations and health care systems around the globe evolved differently. Substantial data come from the German national surveillance network in an outpatient and private practice setting, while information on hospitalized patients in Germany is rather limited.

**Methods:**

Data from the Federal Statistics Office comprising health insurance claims of the entire nationwide inpatient sample from 2005 to 2012 were used to identify patients who were hospitalized for laboratory-confirmed influenza and to analyse demographical aspects, comorbidities, hospitalization duration, outcomes and ventilator use during the pandemic and seasonal waves of influenza.

**Measurements and main results:**

A number of 34,493 admissions for laboratory-confirmed influenza occurred during waves between 2005 and 2012. During the pandemic seasonal waves, the number of hospitalizations vastly surpassed the level that was seen in any of the seasonal waves. A major demographic shift was seen with respect to patient age, as younger patients (< 60 years old) were more frequently hospitalized. Mean length of stay was shorter (149 vs. 193 hours), mean time on ventilation tended to be shorter (261 vs. 305 hours) in young children (< 4 years old) and longer (393 vs. 339 hours) in the elderly (> 60 years old). Time to ventilation was shorter in non-fatal cases (328 vs. 349 hours) and longer in fatal cases (419 vs. 358 hours). Logistic regression was used to show the impact of comorbidities and co-diagnoses on mortality and the need for ventilation, as well as differences between pandemic and seasonal influenza.

**Conclusions:**

Inpatient data suggest differences in patient populations during pandemic and seasonal influenza. Younger patients were more frequently hospitalized. Differences with respect to the presence of certain comorbidities and co-diagnoses, length of stay, time to ventilation and ventilation time could be identified.

## Introduction

Influenza is a viral infectious disease that appears as yearly seasonal epidemics of varying severity[1]. Clinically-relevant morbidity is caused by virus types A and B. Influenza A is further subtyped according to the haemagglutinin (H) and neuraminidase (N) antigens[1]. Influenza has major impact on healthcare systems and economies around the globe[1]. Pandemic waves, i.e., rapid worldwide spreads among large populations, were observed in 1918–1920 (‘Spanish flu’, A/H1N1), 1957 (‘Asian flu’), 1968 (‘Hong Kong flu’), 1977/78 (‘Russian flu’, A/H1N1) with the most recent pandemic occurring in 2009 (‘swine flu’, A/H1N1)[2]. Due to the wide spread of the disease during pandemic waves, a significantly greater morbidity and a higher death toll can be attributed to influenza pandemics as compared to seasonal occurrences of influenza[2]. The first cases of the 2009 pandemic were detected in the Mexican federal district of Mexico City on 18^th^ March, 2009[3–5]. Cases were reported in the southern region of the United States of America by the end of March[6]. The highest infectious counts of H1N1 in different countries occurred non-contemporaneously around the globe, with peak numbers in Germany in November[7].

The available literature on the H1N1 pandemic suggests differences between the pandemic and seasonal waves of former years with respect to spread, demographics, comorbidities, clinical course and outcomes. Likewise, great international variances could be seen in these parameters during the pandemic. In Canada, two distinct waves were reported which differed with respect to demographics and age distribution, underlying conditions, ICU admissions and outcomes[8]. Generally, younger patients were affected at a higher frequency during the H1N1 pandemic[3,9–12]. With respect to outcomes, different findings have been reported. In a study analysing national registry data from France and the United States, younger age could be identified as a principal risk factor for mortality in these countries, as relative mortality was higher in younger age groups[13]. Between April and August of 2009, 1,088 lab-confirmed cases were reported to the California Department of Public Health that resulted in hospitalization or death. The median age of hospitalized patients was younger than commonly anticipated in seasonal influenza[14]. Infants had the highest hospitalization rates and persons aged 50 years or older had the highest mortality rates upon hospitalization. Established risk factors for the potential complications of seasonal influenza were present in most cases. Registry-based surveillance in Denmark revealed that while influenza-associated overall hospitalizations were not increased as compared to previous seasons, there was a disproportionally large impact on the age group 5–24 years [9]. Another study from Australia suggested that pregnancy is a risk factor and is related to more and severe complications (e.g., ICU admission)[15]. A large population-based study of pandemic influenza in the United States identified pregnancy as a risk factor for hospitalization. A review of 332 fatalities in Argentina revealed comorbidities such as obesity and pre-existing pulmonary disease as risk factors for a fatal outcome, as well as pregnancies with a late onset of antiviral treatment[16]. Among 9,966 Chinese patients, the prevalence of chronic medical conditions, pregnancy, or obesity was significantly higher in patients with severe illness than in those with less severe illness [17]. In 112 patients from Melbourne, Australia, pregnancy was a risk factor for hospitalization[18]. This is also implied by an early American study from April to May 2009 on 34 pregnant women with probable or confirmed H1N1 infection[19]. Data obtained by a French institute for public health surveillance from worldwide sources led to a similar conclusion, that pregnant women and those with metabolic comorbidities were at risk for a fatal outcome[20]. A report from New Zealand hinted that ethnicity played a role, since hospitalizations were markedly higher for Māori and Pacific peoples compared with Europeans and others[21]. First Nation descent was likewise identified as a risk factor for severe outcomes (as defined by ICU admission) in a case control study from Manitoba, Canada[22]. Individual patient data of 542 cases from Manitoba County, USA, also suggests non-white ethnicity as a risk factor for in-house mortality, as well as chronic lung disease, a recent history of cancer, immuno-suppression and delay of hospital admission[23]. In-hospital surveillance data on 205 cases from Ireland showed that age-specific hospitalization rates were highest in 15- to 19-year-olds and lowest in those aged 65 years and over. Median length of stay was 24 days. Four hospitalized individuals (2%) died. Asthma, haemoglobinopathies and immunosuppression were the most common comorbidities [24]. An analysis of 440 fatal cases in Great Britain from April 2009 to March 2010 showed that fatal cases were mainly seen in young adults (median age 43 years) with the highest attributable fractions being chronic neurological disease (24%), immunosuppression (16%) and respiratory disease (15%)[25]. In a multivariate analysis, risk factors for disease severity among patients hospitalized for H1N1 in Spain from April to December 2009 were identified as starting antiviral therapy more than 48 hours after symptom onset, morbid obesity, cardiovascular disease and chronic obstructive pulmonary disease[26].

To date, German inpatient data have not been made available at a larger scale. Multicentre data from 15 participating hospitals were aggregated within the pandemic influenza surveillance network (PIKS), but the observation period only spanned the 49^th^ week of 2009 to the 12^th^ week of 2010, unfortunately well beyond the peak influenza wave, so that the majority of hospitalizations were actually missed[27]. In addition, inpatient data were only partially collected, from reports which are made available by the Robert Koch Institute and which rely on the legal requirement to report influenza cases to the departments of health (Infektionsschutzgesetz)[7,28]. Data tend to be incomplete with regard to comorbidities and are not available before the 29^th^ week of 2009[7]. No detailed data on key variables, such as the use of ventilation or length of stay are thus available. This applies even more to seasonal influenza epidemics in former years. We hereby aim to present the data of all hospital admissions in Germany aggregated from nationwide insurance claims.

## Material and methods

### Database

International Classification of Diseases, version 10 (ICD-10) data coded according to G-DRG (German modification of the diagnosis related groups) were obtained from the Federal Statistical Office of Germany (DeStatis). DeStatis holds data on nationwide health insurance claims of the entire inpatient sample. Since there is a legal requirement for healthcare insurance which applies to the vast majority of the German population, the inpatient sample is nearly fully assessed. Data sets comprise detailed pseudonymized information on age, gender, admission and dismissal times, primary diagnoses and 89 co-diagnoses, as well as 100 operation and procedure keys (OPS). OPSs are codes that reflect the German modification of the international classification of procedures in medicine (ICPM), which allow access to information such as time of intubation and hours of mechanical ventilation. Data were aggregated and analysed using SAS 9.4. Where aggregated query data resulted in numbers less than three, data were censored in compliance with the DeStatis anonymization policy.

### Case selection

Patient data sets were selected by screening for influenza-related ICD-Codes, including J09 for pandemic influenza (i.e., influenza due to certain identified influenza viruses including the influenza A/H1N1 pandemic of 2009 known as swine flu) and J10 (i.e., influenza due to another identified influenza virus). Additionally, data sets containing the code U69.20 (a special code for H1N1 influenza) were selected. Where specified, the code J11 (i.e., influenza as a clinical diagnosis although the virus was not identified) was included. J09 and J10 encode laboratory-confirmed influenza. J11 relies upon a clinical diagnosis and therefore largely corresponds to the term ‘influenza-like illness’ (ILI) but does not allow a definite diagnosis. Cases were selected from the time frame of the German influenza wave, largely based on the definition of the German influenza surveillance service (Robert-Koch Institut).

### Statistical analysis

Demographics and descriptive statistics were derived from the above-mentioned data sets to characterize patients with regard to gender, age, comorbidities and co-diagnoses, length of stay, time from admission to intubation and hours of mechanical ventilation. Diagnosis codes are given in S4 Table. Differences between the H1N1 2009 pandemic influenza and seasonally occurring epidemic influenza were highlighted. To further explore differences between pandemic and seasonal influenza concerning underlying comorbidities and co-diagnoses, logistic regression was performed with the presence of pandemic influenza in one patient as the outcome variable. To examine the impact of comorbidities and co-diagnoses on endpoints, such as mortality and the need for mechanical ventilation, several explorative analyses were performed using logistic regression. Length of stay, hours until intubation and hours of mechanical ventilation were compared using the Kruskal-Wallis test followed by the Wilcoxon test. Categorical variables were compared using the chi-squared test. Hospitalizations, use of mechanical ventilation and mortality rates were compared between pandemic and seasonal influenza and odds ratios were calculated. The hospitalization rate per 100,000 individuals per season was calculated using the age- and gender-specific census from 2011, as provided by DeStatis.

## Results

### Overall hospitalizations

**Fig 1:**
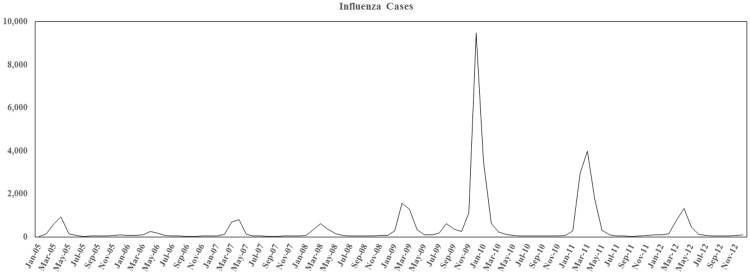
Graphical illustration of the total number of hospitalized cases. Seasonal influenza waves and the H1N1 pdm09 wave, according to the ICD statistics of the German Federal Statistical Office. Cases comprise the ICD codes J09 and J10 representing laboratory-confirmed cases.

Fig 1 shows a graphic representation of influenza waves from 2004/05 until 2011/12. Numbers represent patients hospitalized according to J09/J10 ICD codes (confirmed influenza cases). Marked differences between the seasons from 2004/05 until 2008/09 and the 2009/2010 pandemic can be seen with respect to the overall numbers of hospitalizations and the timing of the main influenza wave. While the highest numbers of hospitalizations due to seasonal influenza waves in Germany are usually to be anticipated between January and March, the 2009 pandemic wave reached its peak markedly earlier, in October and November of 2009. The ICD statistics also suggest a much higher number of influenza-related hospitalizations for pandemic influenza as compared to previous seasonal outbreaks.

### Age and gender distribution of hospitalized cases

**Fig 2:**
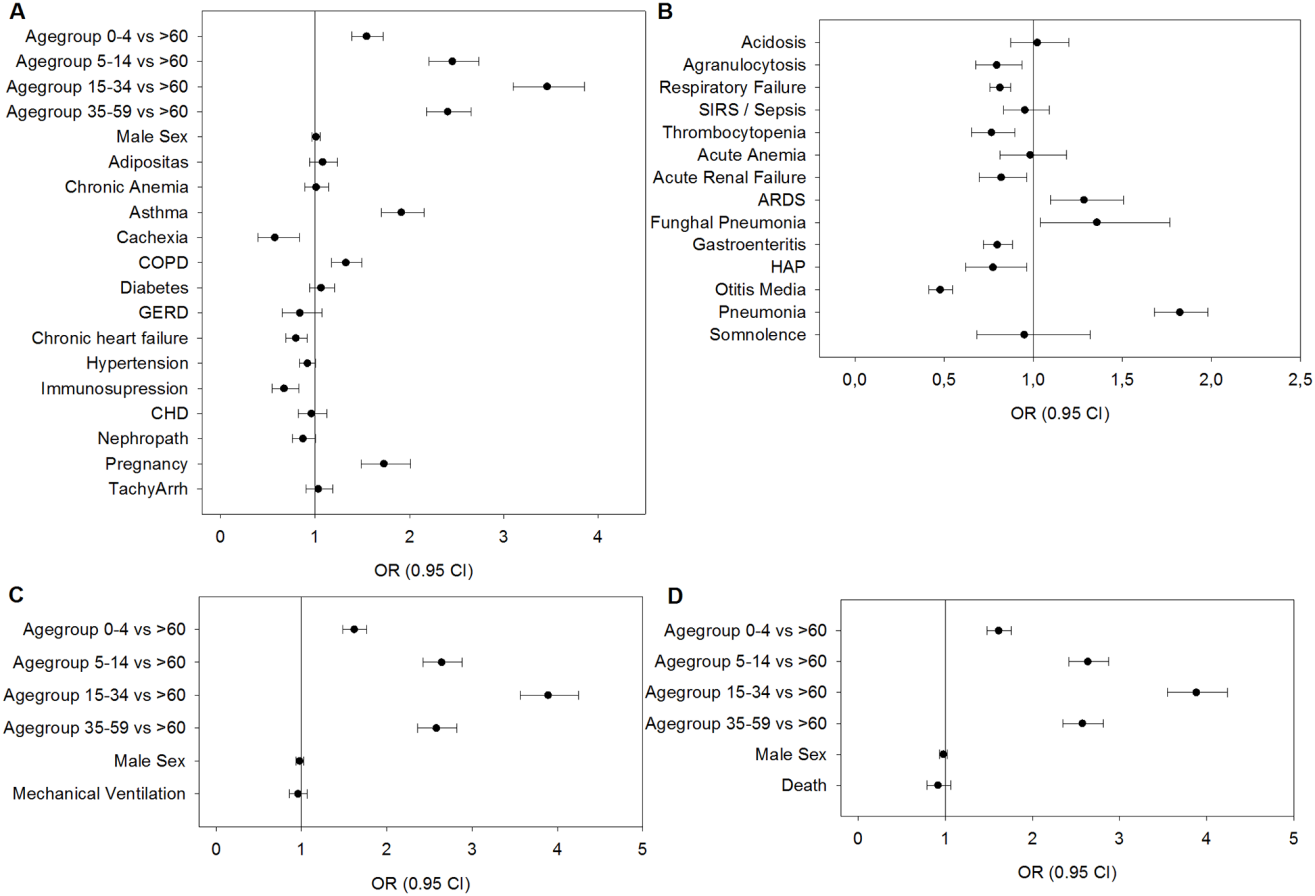
Odds ratios of pandemic vs. seasonal influenza. Logistic regression was used to estimate the difference of pandemic and seasonal influenza regarding age, gender, death, mechanical ventilation, and certain co-diagnoses. Presence of pandemic influenza was set as the outcome variable. ORs of greater than 1 meaning the analysed factor occurring favourably in pandemic influenza cases. (A) ORs for pre-existing comorbidities, age and gender, (B) OR for co-diagnoses and complications. (C) ORs for mechanical ventilation and age, (D) ORs for death and age. ORs and their respective 95% confidence intervals are determined by logistic regression. Pandemic influenza was set as the outcome, therefore ORs greater than 1 favour pandemic over seasonal influenza.

**Fig 3:**
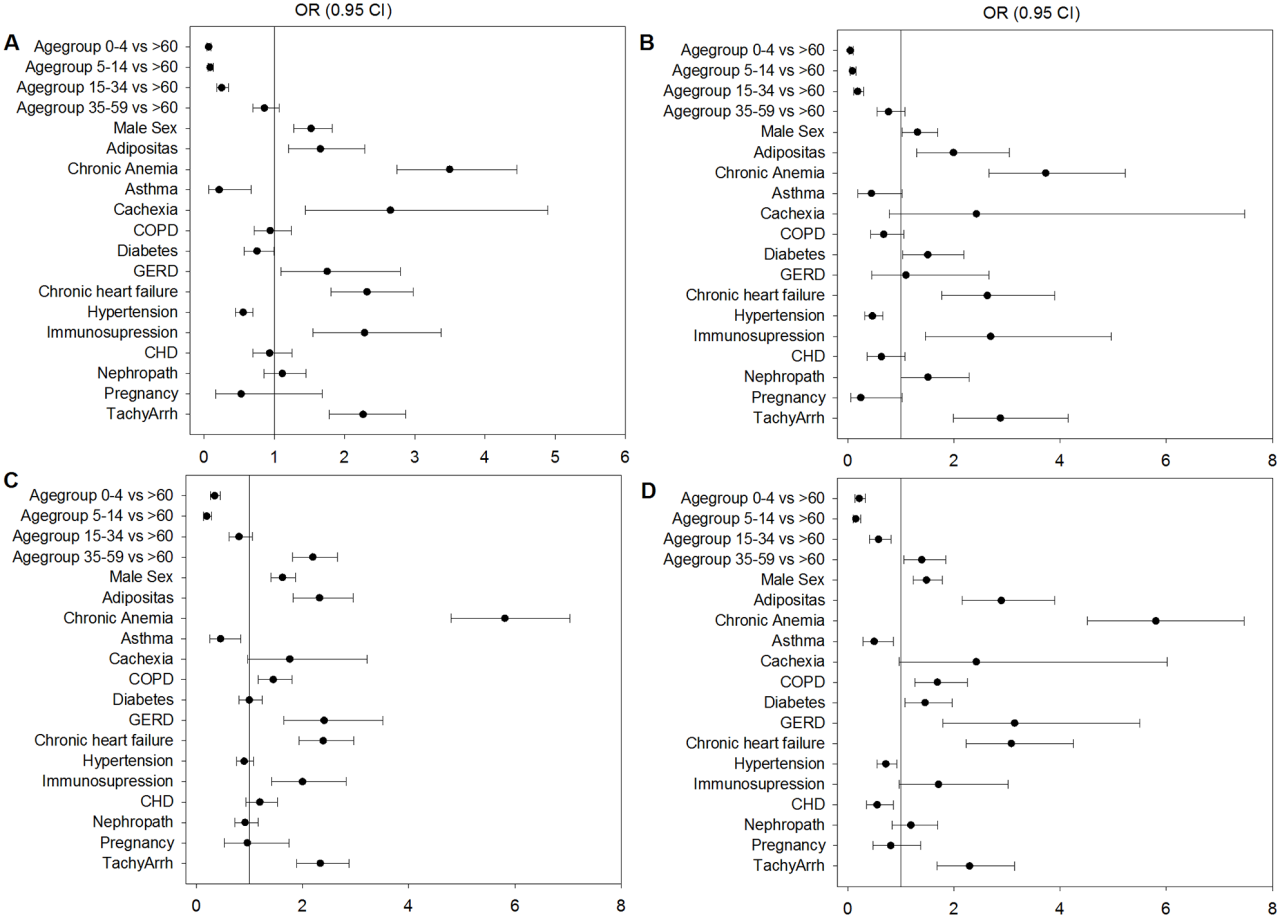
Influence of comorbidities and long-standing diagnoses on the outcome of mechanical ventilation and death in seasonal and pandemic influenza. Logistic regression was used to estimate the influence of certain variables, such as age, gender and comorbidities on outcomes of mechanical ventilation and death. (A) Influence of comorbidities on fatal outcomes in seasonal influenza. ORs greater than 1 mean that the analysed factor occurred favourably in fatal cases. (B) Influence of comorbidities on fatal outcomes in pandemic influenza. ORs greater than 1 mean that the analysed factor occurred favourably in fatal cases. (C) Influence of comorbidities on the outcome of MV in seasonal influenza. ORs greater than 1 mean that the analysed factor occurred favourably in MV cases. (D) Influence of comorbidities on the outcome of MV in pandemic influenza. ORs greater than 1 mean that the analysed factor occurred favourably in MV cases.

**Fig 4:**
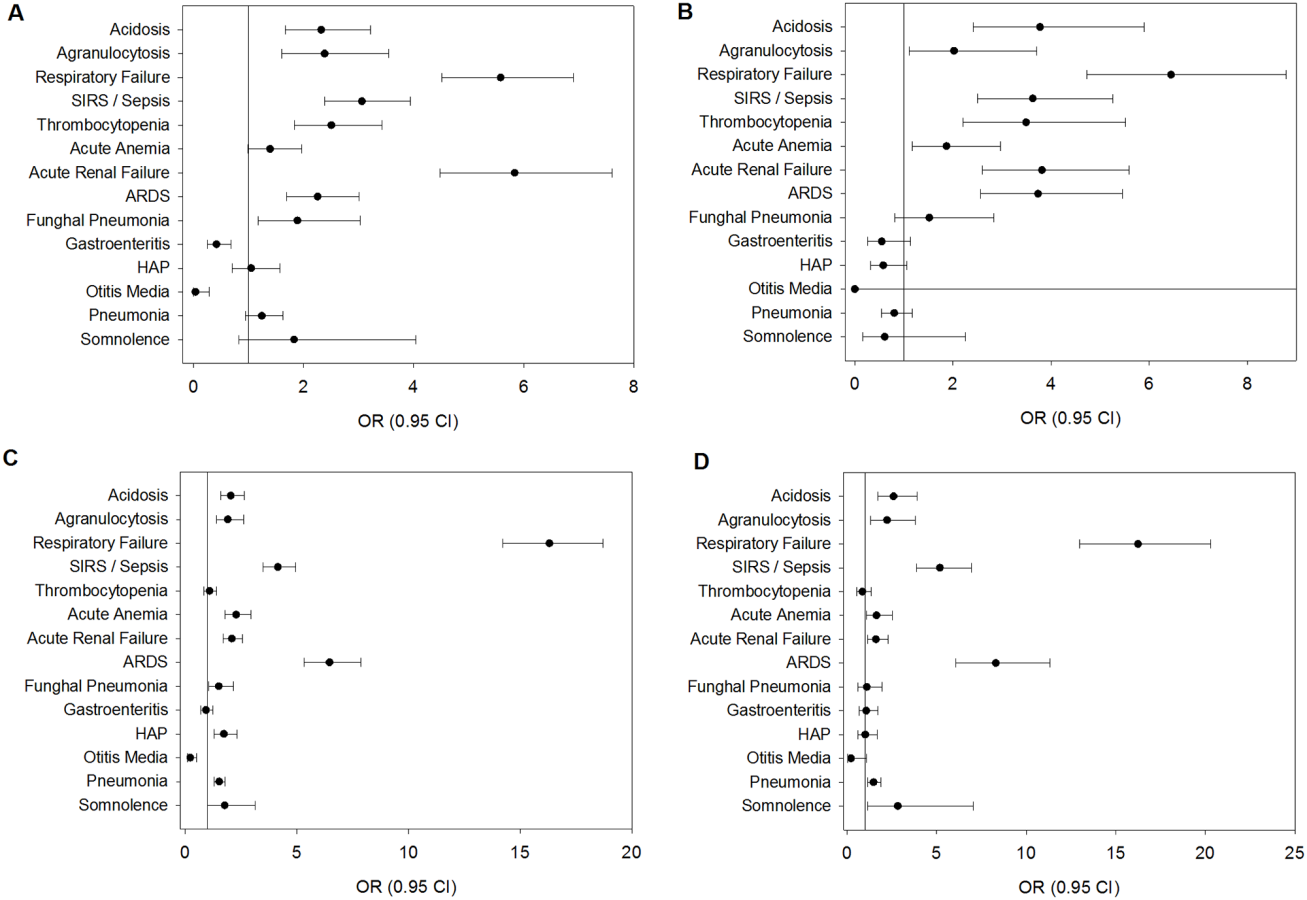
Influence of co-diagnoses and complications on the outcome of mechanical ventilation and death in seasonal and pandemic influenza. Logistic regression was used to estimate the influence of certain variables, such as age, gender and co-diagnoses on outcomes of mechanical ventilation and death. (A) Influence of co-diagnoses on fatal outcomes in seasonal influenza. ORs greater than 1 mean that the analysed factor occurred favourably in fatal cases. (B) Influence of co-diagnoses on fatal outcomes in pandemic influenza. ORs greater than 1 mean that the analysed factor occurred favourably in fatal cases. (C) Influence of co-diagnoses on the outcome of MV in seasonal influenza. ORs greater than 1 mean that the analysed factor occurred favourably in MV cases. (D) Influence of co-diagnoses on the outcome of MV in pandemic influenza. ORs greater than 1 mean that the analysed factor occurred favourably in MV cases.

As outlined in the introduction, studies from other countries and previous reports from Germany’s legal reporting system for influenza cases indicate that an unanticipatedly high caseload of influenza was seen in patients younger than 60 years old with pandemic influenza as compared to seasonal influenza. Table 1 shows the distribution of cases among patients hospitalized for seasonal and 2009/10 pandemic influenza in Germany. Hospitalization rates among all age groups are higher during the pandemic. Yet the age-specific hospitalization rates of patient groups younger than 60 years old are higher than in seasonal influenza; accordingly, patients older than 60 are less frequently hospitalized, even after consideration of the higher overall hospitalization rate. Unadjusted odds suggest that very young patients (0–4 years old) and older patients (> 60 years old) are comparably less frequently hospitalized (Table 1). Logistic regression confirms this view (Figs 2–4, see below). The odds of hospitalization show a slight preference for the female sex in pandemic influenza. Logistic regression suggests that after adjustment for other factors, such as comorbidities, neither the male nor female sex is preferably hospitalized in pandemic influenza as opposed to seasonal influenza (Fig 2A).

**Table 1:** Hospitalizations, mechanical ventilation and fatal cases in seasonal influenza waves and the 2009 pandemic.

**Hospitalizations**						
	**Seasonal Flu**		**Pandemic Flu**		**Pandemic vs. Seasonal**	
**Gender**	**N (% All)**	**Hospitalization Rate**	**N (% All)**	**Hospitalization Rate**	**unadj. OR (CI)**	**p-value**
**Female**	9,704 (46.6)	3.38	6,554 (47.9)	15.96	1.05 (1.01–1.1)	0.0235
**Male**	11,102 (53.4)	4.05	7,133 (52.1)	18.22	0.95 (0.91–0.99)	
**Age Group**						
**0–4**	6,328 (30.4)	27.07	2,989 (21.8)	89.52	0.64 (0.61–0.67)	< 0.0001
**5–14**	4,623 (22.2)	8.85	3,566 (26.1)	47.76	1.23 (1.17–1.3)	< 0.0001
**15–34**	3,078 (14.8)	2.38	3,500 (25.6)	18.95	1.98 (1.87–2.09)	< 0.0001
**35–59**	3,614 (17.4)	1.74	2,711 (19.8)	9.12	1.17 (1.11–1.24)	< 0.0001
**> 60**	3,163 (15.2)	2.13	921 (6.7)	4.34	0.40 (0.37–0.43)	< 0.0001
**All**	20,806 (100)	3.71	13,687 (100)	17.06		
**Mechanical Ventilation**						
	**N (% All)**					
	**Seasonal Flu**		**Pandemic Flu**		**Pandemic vs. Seasonal**	
**Gender**	**N (% All)**	**MV Rate (%)**	**N (% All)**	**MV Rate (%)**	**unadj. OR (CI)**	**p-value**
**Female**	651 (63.2)	6.71	356 (58.1)	5.43	0.8 (0.7–0.91)	0.0009
**Male**	379 (36.8)	3.41	257 (41.9)	3.6	1.06 (0.89–1.25)	0.4968
**Age Group**						
**0–4**	100 (9.7)	1.58	39 (6.4)	1.3	0.82 (0.57–1.2)	0.3059
**5–14**	39 (3.8)	0.84	31 (5.1)	0.87	1.03 (0.64–1.66)	0.9003
**15–34**	118 (11.5)	3.83	125 (20.4)	3.57	0.93 (0.72–1.2)	0.5737
**35–59**	463 (45)	12.81	302 (49.3)	11.14	0.85 (0.73–0.99)	0.0436
**> 60**	310 (30.1)	9.8	116 (18.9)	12.6	1.33 (1.06–1.66)	0.0146
**All**	1,030 (100)	4.95	613 (100)	4.48	0.9 (0.81–1)	0.0441
**Fatal Cases**						
	**N (% All)**					
	**Seasonal Flu**		**Pandemic Flu**		**Pandemic vs. Seasonal**	
**Gender**	**N (% All)**	**Fatality Rate (%)**	**N (% All)**	**Fatality Rate (%)**	**unadj. OR (CI)**	**p-value**
**Female**	362 (62.5)	3.73	168 (57.3)	2.56	0.68 (0.56–0.82)	<0.0001
**Male**	217 (37.5)	1.95	125 (42.7)	1.75	0.89 (0.72–1.12)	0.326
**Age Group**						
**0–4**	26 (4.5)	0.41	9 (3.1)	0.3	0.73 (0.34–1.56)	0.4188
**5–14**	24 (4.1)	0.52	18 (6.1)	0.5	0.97 (0.53–1.79)	0.928
**15–34**	48 (8.3)	1.56	37 (12.6)	1.06	0.67 (0.44–1.04)	0.0719
**35–59**	219 (37.8)	6.06	145 (49.5)	5.35	0.88 (0.71–1.09)	0.2294
**> 60**	262 (45.3)	8.28	84 (28.7)	9.12	1.11 (0.86–1.44)	0.422
**All**	579 (100)	2.78	293 (100)	2.14	0.76 (0.66–0.88)	0.0002

The upper panel shows total hospitalizations (N) for different age groups and both sexes. Age and gender proportions are given in parentheses (% of all). The hospitalization rate is given as cases per 100,000 persons per season, of each respective subpopulation. The unadjusted odds (OR) of hospitalization are given to compare seasonal and pandemic influenza. P-values and 95% confidence intervals are given. The middle panel shows the absolute number of patients requiring mechanical ventilation (MV). The age- and gender-specific proportions are given in parentheses (% of all). The age- and gender-specific rates of mechanical ventilation (MV rate) were calculated in relation to the respective group of all hospitalizations. The given odds ratios compare age- and gender-specific MV rates. P-values and 95% confidence intervals are given. The lower panel shows the absolute number of fatal cases for different age groups and both sexes (N). Age- and gender-specific proportions among fatal cases are given in parentheses (% of all). The age- and gender-specific fatality rate is given. The given odds ratios are to compare age- and gender-specific fatality rates. P-values and 95% confidence intervals are given.

### Age and gender distribution among cases of mechanical ventilation

As shown in Table 1, gender-specific proportions among ventilated patients show a preference for the male sex in pandemic influenza (proportions of 41.9 vs. 36.8%). Likewise, younger patient groups < 60 years old, and most prominently those between 15–35 and 35–59 years old require mechanical ventilation (MV) more frequently during pandemic influenza rather than seasonal influenza. Yet, with respect to hospitalized patients, the age- and gender-specific rates of mechanical ventilation show that in fact older patients (> 60 years old) have higher odds of MV in pandemic influenza. Younger age groups either do not show differences in MV rates or have lower MV rates (particularly those between 35 and 59 years old). Female patients have significantly lower odds of MV in pandemic influenza. Higher frequencies or proportions of MV are thus most likely explained by the higher hospitalization rates of specific subgroups. The overall rate of mechanical ventilation in patients does not appear to be higher in pandemic influenza (4.48%) as compared to seasonal influenza (4.95%), and the OR of pandemic influenza vs. seasonal influenza is 0.9. This is confirmed by logistic regression upon age adjustment (Fig 2C).

### Gender- and age-specific mortality among hospitalized cases

A similar distribution in age and gender proportions, as seen in hospitalizations and use of MV, can be observed in the in-hospital mortality rate of influenza cases (Table 1). Age- and gender-specific mortality is not higher in younger patients in pandemic influenza than would be anticipated from the data on seasonal influenza waves. Overall in-hospital mortality is rather lower in pandemic influenza. Logistic regression confirms that age-adjusted overall mortality remains at the same levels as seen in seasonal influenza (Fig 2B).

### Time until usage of ventilation

The mean time from hospital admission until the start of ventilation is not significantly different between seasonal and pandemic influenza except for the group of older patients. However, there is a trend towards earlier intubation (140 vs. 174 hours, Table 2). This difference is more pronounced in fatal cases (Table 2, S2 Table) and varies among age groups.

**Table 2:** Length of stay, time until intubation and hours of ventilation.

	Length of Stay			Time until Intubation			Hours of Ventilation		
**Gender**	**Seasonal Flu**	**Pandemic Flu**	**p-value**	**Seasonal Flu**	**Pandemic Flu**	**p-value**	**Seasonal Flu**	**Pandemic Flu**	**p-value**
**Female**	185/114 (± 262)	146/87 (± 219)	<.0001	176/43 (± 380)	126/44 (± 214)	0.9225	342/216 (± 446)	335/202 (± 371)	0.7709
**Male**	199/111 (± 329)	153/82 (± 264)	<.0001	173/45 (± 371)	151/31 (± 439)	0.3126	363/233 (± 442)	364/217 (± 481)	0.3239
**Age Group**									
**0–4**	140/97 (± 246)	118/83 (± 171)	<.0001	316/63 (± 734)	173/85 (± 267)	0.8326	305/183 (± 419)	261/196 (± 211)	0.3064
**5–14**	115/75 (± 184)	94/67 (± 122)	<.0001	113/17 (± 285)	157/74 (± 219)	0.0614	256/129 (± 309)	264/160 (± 364)	0.5976
**15–34**	158/93 (± 263)	122/72 (± 200)	<.0001	150/35 (± 283)	108/21 (± 232)	0.1529	353/192 (± 374)	328/215 (± 359)	0.4178
**35–59**	288/150 (± 421)	232/126 (± 361)	<.0001	140/32 (± 284)	147/44 (± 455)	0.1995	384/260 (± 514)	368/229 (± 489)	0.1721
**> 60**	339/240 (± 323)	325/207 (± 351)	<.0001	198/70 (± 352)	142/48 (± 246)	0.0381	339/223 (± 363)	393/209 (± 439)	0.8203
**Fatal Cases**	513/348 (± 626)	484/331 (± 503)	<.0001	235/70 (± 417)	177/57 (± 300)	0.1982	368/245 (± 506)	419/289 (± 452)	0.2070
**Non-Fatal Cases**	184/111 (± 280)	142/83 (± 229)	<.0001	147/35 (± 350)	127/34 (± 382)	0.9857	349/221 (± 416)	328/196 (± 430)	0.0882
**All**	193/113 (±300)	149/85 (±243)	<.0001	174/44 (± 374)	140/37 (± 362)	0.3736	355/227 (± 444)	352/212 (± 438)	0.3182

The left section of the table compares the age- and gender-specific length of hospital stay between pandemic and seasonal influenza. The middle section of the table compares the time until intubation. The right part of the table compares hours of ventilation. Data are given in mean/median hours ± standard deviation. Numbers were rounded to full hours. The Wilcoxon test was used to compare age- and gender-specific length of stay, time until intubation and hours of ventilation.

### Time of mechanical ventilation

Overall, mean time (in hours) of mechanical ventilation per case do not differ in pandemic and in seasonal influenza (Table 2), though ventilation times are generally longer in fatal cases and shorter in non-fatal cases, depending on the selected age group (see S3 Table).

### Length of stay

The average length of stay per case is shorter in pandemic influenza than in seasonal influenza (Table 2). This trend is more pronounced in non-fatal cases with more similarity in fatal cases, except for very young patients (0–4 years old) (see S1 Table).

### Comorbidities and complications of hospitalized cases

#### Comorbidities and complications impacting mechanical ventilation and fatal outcomes

As depicted in Fig 3A–3D, logistic regression suggests that comorbid conditions including tachyarrhythmia, immunosuppression, chronic obstructive pulmonary disease, chronic heart failure, chronic anaemia, body weight disorders (especially obesity), male sex and older age are risk factors either for fatal outcomes (Fig 3A and 3B), mechanical ventilation (Fig 3C and 3D) or both in pandemic and seasonal influenza. Diabetes may be another risk factor for both outcomes in pandemic influenza (Fig 3B and 3D). Younger age is generally protective with regard to these outcomes in seasonal and pandemic influenza.

Fig 4A–4D shows the impact of co-diagnoses and complications on fatal outcomes (Fig 4A and 4B) and mechanical ventilation (Fig 4C and 4D) as estimated by logistic regression. Adult respiratory distress syndrome (ARDS), acute renal injury, acute anaemia, thrombocytopenia, SIRS or Sepsis, respiratory failure, agranulocytosis and acidosis seem to be associated with fatal outcomes in seasonal and pandemic influenza. Hospital-acquired pneumonia alone does not appear to be an independent predictor of mortality (Fig 4A and 4B) in this model but seems to be associated with mechanical ventilation (Fig 4C and 4D), while thrombocytopenia is not significantly associated with mechanical ventilation. The other complications tend to have similar relevance to mechanical ventilation as to mortality, given individual differences in effect sizes. Fungal pneumonia is a significant complication for both outcomes in seasonal influenza (Fig 4A and 4C) but not in pandemic influenza (Fig 4B and 4D).

#### Differences concerning comorbidities and co-diagnoses

**Fig 5:**
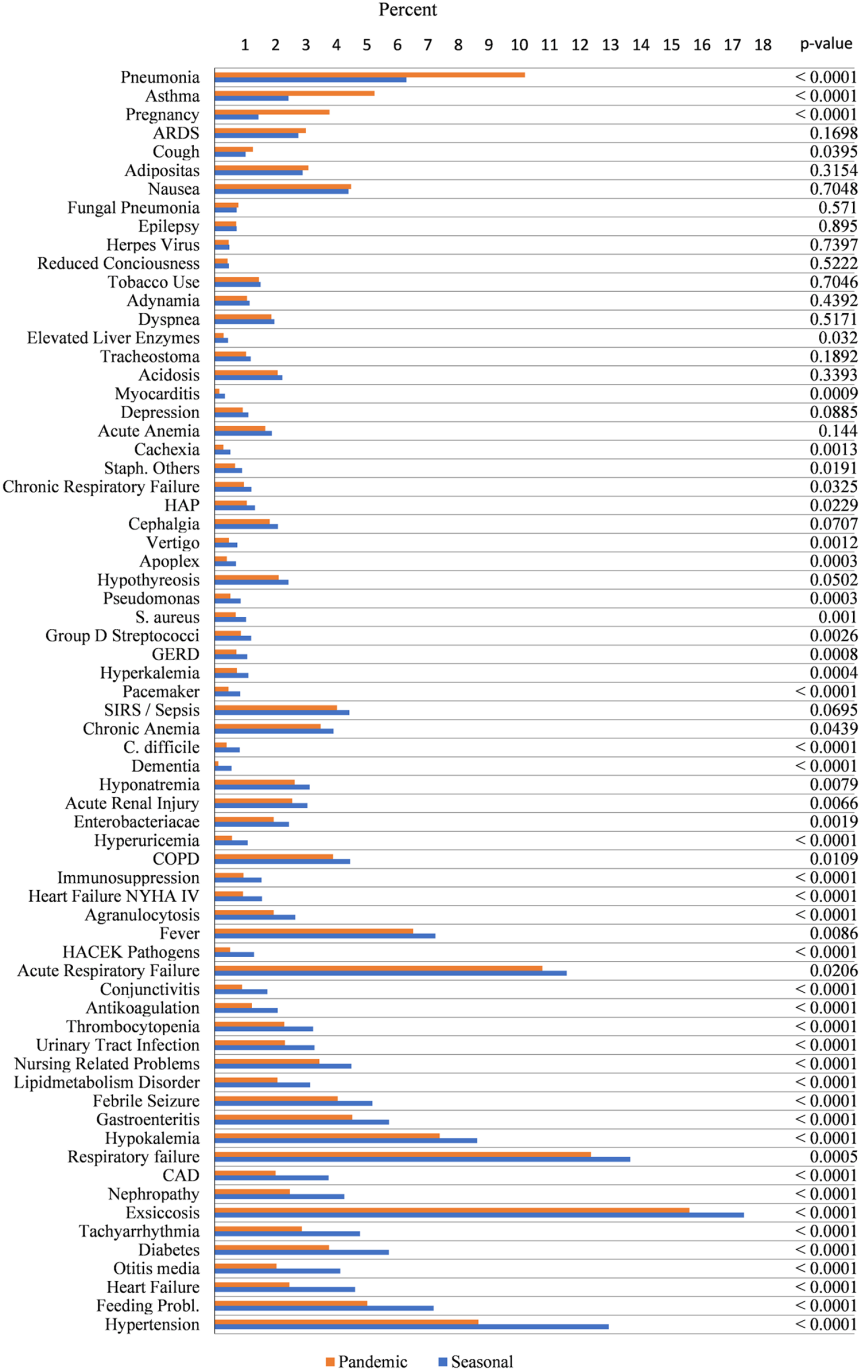
Relative frequencies of comorbidities and co-diagnoses in pandemic and seasonal influenza. Relative frequencies are given as a percentage of all hospitalized patients. Comorbidities are ordered by the magnitude of the absolute difference in relative frequencies between pandemic (red) and seasonal influenza (blue). Comorbidities that number less than three are censored in compliance with the DeStatis data protection policy.

**Fig 6:**
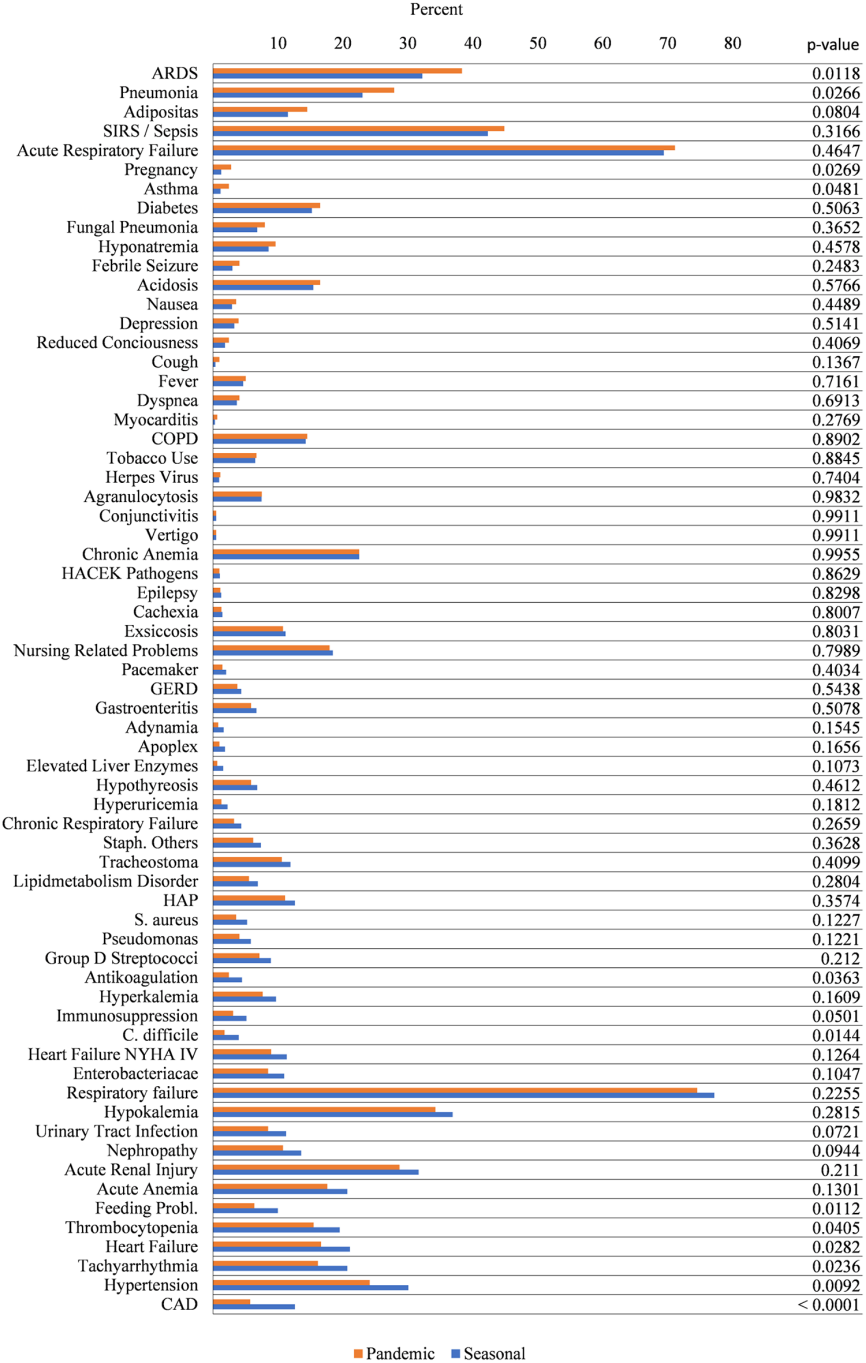
Relative frequencies of comorbidities and co-diagnoses in patients receiving mv in pandemic and seasonal influenza. Relative frequencies are given as a percentage of all hospitalized patients receiving MV. Comorbidities are ordered by the magnitude of the absolute difference in relative frequencies between pandemic (red) and seasonal influenza (blue). Comorbidities that number less than three are censored in compliance with the DeStatis data protection policy.

**Fig 7:**
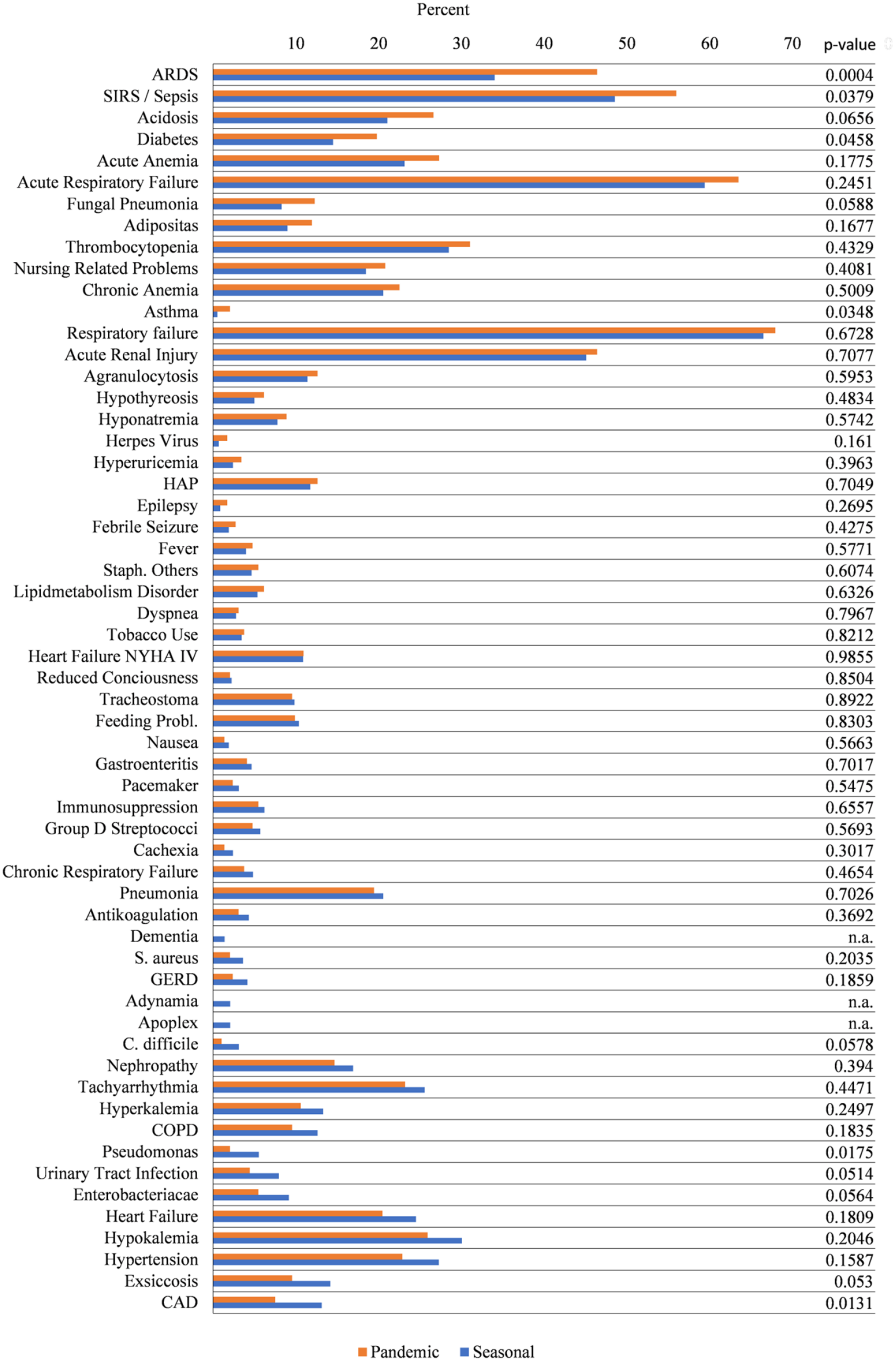
Relative frequencies of comorbidities and co-diagnoses in fatal cases of pandemic and seasonal influenza. Relative frequencies are given as a percentage of all fatal cases. Comorbidities are ordered by the magnitude of the absolute difference in relative frequencies between pandemic (red) and seasonal influenza (blue). Comorbidities that number less than three are censored in compliance with the DeStatis data protection policy.

Fig 5 shows the percentages of comorbidities and co-diagnoses in seasonal and pandemic influenza. Subgroups of patients receiving MV (Fig 6) and fatal cases (Fig 7) are analysed separately. Looking at the entire samples of hospitalized patients, only a minor part of comorbidities and co-diagnoses are relatively more frequent in the pandemic cases, e.g., pneumonia, pregnancy, ARDS and asthma (but not COPD) (Fig 5). Many comorbidities that are typically related to advanced age are relatively more frequent in seasonal influenza (e.g., cardiac, vascular, cerebral, or metabolic disease). ARDS, pneumonia, pregnancy and asthma seem to be more frequent among ventilated patients with pandemic influenza (Fig 6). Co-diagnoses indicating a severe course of disease (e.g., ARDS, SIRS or Sepsis, fungal pneumonia) are more frequent among fatal cases of pandemic influenza (Fig 7). For further reference, S5 Table contains all underlying data regarding absolute and relative frequencies of comorbidities. Fig 2A summarizes the results of logistic regression analyses, which investigated whether certain comorbidities are associated with the 2009 pandemic influenza rather than the seasonal influenza. Thus, age-adjusted odds ratios greater than 1 favour pandemic influenza over seasonal influenza. Interestingly, respiratory comorbidities such as asthma and COPD seem to be risk factors for pandemic influenza in this model. Pregnancy also seems to be associated with pandemic influenza cases. Chronic heart failure, immunosuppression and low body weight (but not adipositas), on the other hand, are associated with seasonal influenza. Other comorbidities, including renal disease, tachyarrhythmia, coronary artery disease, GERD, diabetes and chronic anaemia do not seem to occur more frequently in either type of influenza. There is also no preference for any sex. Younger age groups have a higher likelihood to be among pandemic influenza cases. Fig 2B points at co-diagnoses that could be associated with pandemic rather than seasonal influenza, as calculated by logistic regression. Odds ratios greater than 1 are in favour of the 2009 pandemic influenza. Respiratory co-diagnoses, such as pneumonia, fungal pneumonia and adult respiratory distress syndrome are more likely to be associated with pandemic influenza cases. Exceptions to these are hospital-acquired pneumonias and acute respiratory failure. Complications like otitis media or gastroenteritis are more frequently seen in seasonal influenza cases. This is also true for acute renal failure and thrombocytopenia, while acute anaemia and agranulocytosis and sepsis do not favour pandemic or seasonal influenza. Logistic regression suggests that bacterial germs, probably in part associated with HAP (pseudomonas, S. aureus) and/or antibiotic treatment (C. difficile) are more frequently associated with seasonal influenza cases (S5 Table). After all, differences between pandemic and seasonal influenza are seen with respect to unadjusted relative frequencies of comorbidities and co-diagnoses, and differences are still suggested after adjustment by logistic regression.

## Discussion and conclusion

The G-DRG data suggest that the 2009/10 pandemic influenza has distinct characteristics when compared to seasonal influenza waves. The study allows for the identification of comorbidities and co-diagnoses that are more frequently associated either with pandemic or seasonal influenza, and can identify their impact on outcomes such as in-hospital mortality and the need for mechanical ventilation. Based upon statutory reports, Buda et al. reported a number of n = 7,882 influenza hospitalizations from the 18^th^ week of 2009 until the 17^th^ week of 2010. This is a considerably lower count than estimated from the G-DRG database (Fig 1, Table 1). Obvious differences with respect to the data sources can be explained by the legal requirement to report an influenza case (or suspicion of one) and the G-DRG coding of cases for the purpose of health insurance claims. In general, the number of reported cases is considered an underestimation of the actual count, and strongly depends on the legal and medical infrastructure of the reporting country. The G-DRG database represents a retrospective cohort and data monitoring is not performed as in prospective cohorts. Nonetheless, data quality is ensured since diagnosis and coding quality is monitored by the professional medical service to the statutory health insurance, which reviews roughly 10% of all insurance claims[29]. Thus, the G-DRG data indicate that the burden of influenza-related hospitalization in Germany may be higher than estimated by the data from the statutory reporting system alone.

A considerable age shift in individuals affected by different types of influenza has been previously reported and can be confirmed using the G-DRG data[28]. Younger patients were more frequently hospitalized during the 2009/10 influenza pandemic, with only very young patients (0–4 years old) as an exception to this[14]. Younger patients were more frequently mechanically ventilated and more often had a fatal outcome in pandemic influenza in total, but not in relation to the hospitalized population. This is in accordance with previously published data[13]. Yet age- and gender-specific MV rates were not elevated in young patients and were in fact higher in older patients aged 60 and over. Overall, mortality rates and rates of mechanical ventilation were not elevated when pandemic influenza is compared to seasonal influenza in this cohort of hospitalized patients. Yet when fatal and non-fatal cases are compared separately, a longer duration of mechanical ventilation and a shorter time until intubation, especially in younger patients, are observed in fatal cases of pandemic influenza, although this is not statistically significant. Pre-existing respiratory diagnoses, including COPD and asthma, and co-diagnoses such as ARDS or pneumonia may be associated with pandemic influenza. Taken together, the data support previously-reported findings that pandemic influenza may have a more severe course of disease in a subset of patients[14,18,20,28,30]. The length of stay was found to be shorter in pandemic influenza. This was consistently seen in fatal and non-fatal cases and across age groups and genders. One explanation may be an earlier and broader administration of anti-viral treatment due to increased awareness towards influenza, especially in 2009. On the other hand, increased demand of hospital resources might have led to an increased patient turn-over and reduced overall length of stay. Pregnancy has been previously reported to be an important risk factor for the 2009 pandemic influenza. Explorative analyses of the G-DRG data hint in this direction. As reported by others, pregnancy seems to be rather associated with pandemic influenza than with seasonal influenza[31]. Logistic regression does not indicate that it increases the odds of worse outcomes such as mechanical ventilation or death, although some reports point towards complications in pregnant patients[15,18–20,32,33].

This study describes differences between seasonally circulating and pandemic influenza (H1N1). However, after the 2009 pandemic, H1N1 appeared also as a seasonal strain. It is believed that when the novel form of H1N1 arose in 2009, it encountered a global population with only limited immunity (older patients) or no immunity (younger patients)[3,12,30,34]. It thus caused a pandemic spread and displayed the distinguishing features detailed here and in previous studies. Older patients may have experienced contact with H1N1 previously, while young patients had not. Since then, the pandemic 2009 H1N1 strain has continued circulating. This study cannot distinguish pathogen- or strain-specific aspects that that affect hospitalization hospitalization from other epidemiological factors (i.e., the environment, climate or travel).

Limitations to this study arise from the fact that the data regarding all diagnoses and procedures were primarily collected in order to facilitate the processing of insurance claims. Bias might have therefore been introduced with respect to the selection of certain diagnoses or co-diagnoses. Confidence, on the other hand, comes from the fact that important findings from this study accord with the reports by investigators from other countries.

Taken together, these results represent a source of information for the preparation against future waves of influenza. Populations that are typically less affected by seasonal influenza, such as younger patients, should particularly be the focus of preventive measures. However, the well-established respiratory comorbidities, such as asthma and COPD, also show a stronger association with pandemic than seasonal influenza. Vaccination strategies should particularly target these risk groups. To our knowledge, this is the first study to evaluate large-scale German in-hospital data and is therefore a valuable supplement to the previously published sentinel network data and statutory reporting system data.

## Supporting information

S1 Table Length of stay. Data are given as mean/median hours (± standard deviation) for fatal and non-fatal cases of seasonal and pandemic influenza.
(DOCX)

S2 Table Time to intubation. Data are given as mean/median hours (± standard deviation) for fatal and non-fatal cases of seasonal and pandemic influenza.
(DOCX)

S3 Table Hours of mechanical ventilation. Data are given as mean/median hours (± standard deviation) for fatal and non-fatal cases of seasonal and pandemic influenza.
(DOCX)

S4 Table ICD codes and respective diagnoses. This table shows the underlying ICD codes for certain diagnoses (comorbidities) as used in S5 Table and Figs 2–4.
(DOCX)

S5 Table Absolute and relative frequencies of comorbidities and co-diagnoses as retrieved from the destatis database. The absolute and relative frequencies of diagnoses in patients with seasonal and pandemic influenza are displayed. In addition, absolute and relative frequencies are shown for subpopulations of mechanically ventilated and non-ventilated patients as well as fatal and non-fatal cases. The chi-squared test was used to compare differences between seasonal and pandemic influenza (all patients) and the according p-values are stated. Empty spaces refer to counts of less than 3 and are censored in compliance with the DeStatis data protection policy.
(DOCX)
