# Supplementary material for: Characteristics and outcomes of a cohort hospitalized for pandemic and seasonal influenza in Germany based on nationwide inpatient data
Source: PLoS One. 2017 Jul 14;12(7):e0180920. doi: 10.1371/journal.pone.0180920 (PMC5510816; doi:10.1371/journal.pone.0180920)
Supplement: S1 Table — Data are given as mean/median hours (± standard deviation) for fatal and non-fatal cases of seasonal and pandemic influenza. (DOCX) [file pone.0180920.s001.docx]

|  | **Non-Fatal** | | **Fatal** | |
| --- | --- | --- | --- | --- |
|  | **Seasonal Flu** | **Swine Flu** | **Seasonal Flu** | **Swine Flu** |
|  |  |  |  |  |
| **Age Group** |  |  |  |  |
| 0 - 4 | 137/97 (± 230) (n=6.302) | 118/83 (± 170) (n=2.980) | 757/416 (± 1251) (n=26) | 300/291 (± 286) (n=9) |
| 5 - 14 | 114/75 (± 184) (n=4.599) | 92/67 (± 109) (n=3.548) | 159/95 (± 193) (n=24) | 448/105 (± 699) (n=18) |
| 15 - 34 | 152/92 (± 256) (n=3.030) | 119/71 (± 193) (n=3.463) | 506/388 (± 441) (n=48) | 451/339 (± 447) (n=37) |
| 35 - 59 | 270/145 (± 388) (n=3.395) | 219/123 (± 350) (n=2.566) | 558/374 (± 710) (n=219) | 468/360 (± 460) (n=145) |
| > 60 | 326/237 (± 301) (n=2.901) | 302/197 (± 313) (n=837) | 485/346 (± 488) (n=262) | 554/342 (± 564) (n=84) |
| **All** | 184/111 (± 280) | 142/83 (± 229) | 513/348 (± 626) | 484/331 (± 503) |
