# Supplementary material for: Characteristics and outcomes of a cohort hospitalized for pandemic and seasonal influenza in Germany based on nationwide inpatient data
Source: PLoS One. 2017 Jul 14;12(7):e0180920. doi: 10.1371/journal.pone.0180920 (PMC5510816; doi:10.1371/journal.pone.0180920)
Supplement: S2 Table — Data are given as mean/median hours (± standard deviation) for fatal and non-fatal cases of seasonal and pandemic influenza. (DOCX) [file pone.0180920.s002.docx]

**Supporting Table 2 (Time to Intubation)**

|  | **Non-Fatal** | | **Fatal** | |
| --- | --- | --- | --- | --- |
|  | **Seasonal Flu** | **Swine Flu** | **Seasonal Flu** | **Swine Flu** |
| **Age Group** |  |  |  |  |
| 0 - 4 | 286/57 (± 705) (n=83) | 199/128 (± 286) (n=32) | 467/68 (± 868) (n=17) | 54/18 (± 94) (n=7) |
| 5 - 14 | 168/27 (± 353) (n=24) | 146/74 (± 200) (n=25) | 24/12 (± 36) (n=15) | 207/98 (± 304) (n=6) |
| 15 - 34 | 122/29 (± 244) (n=90) | 86/22 (± 176) (n=106) | 241/73 (± 374) (n=28) | 230/19 (± 412) (n=19) |
| 35 - 59 | 103/24 (± 219) (n=333) | 139/37 (± 511) (n=216) | 234/59 (± 391) (n=130) | 170/67 (± 267) (n=86) |
| > 60 | 173/59 (± 328) (n=174) | 110/29 (± 158) (n=66) | 229/99 (± 379) (n=135) | 183/62 (± 326) (n=50) |
| **All** | 147/35 (± 350) | 127/34 (± 382) | 235/70 (± 417) | 177/57 (± 300) |
