# Supplementary material for: Characteristics and outcomes of a cohort hospitalized for pandemic and seasonal influenza in Germany based on nationwide inpatient data
Source: PLoS One. 2017 Jul 14;12(7):e0180920. doi: 10.1371/journal.pone.0180920 (PMC5510816; doi:10.1371/journal.pone.0180920)
Supplement: S3 Table — Data are given as mean/median hours (± standard deviation) for fatal and non-fatal cases of seasonal and pandemic influenza. (DOCX) [file pone.0180920.s003.docx]

|  | **Non-Fatal** | | **Fatal** | |
| --- | --- | --- | --- | --- |
|  | **Seasonal Flu** | **Swine Flu** | **Seasonal Flu** | **Swine Flu** |
|  |  |  |  |  |
| **Age Group** |  |  |  |  |
| 0 - 4 | 284/180 (± 380) (n=83) | 257/190 (± 204) (n=32) | 435/244 (± 605) (n=17) | 289/222 (± 276) (n=7) |
| 5 - 14 | 286/173 (± 334) (n=24) | 203/153 (± 182) (n=25) | 175/89 (± 215) (n=15) | 598/199 (± 774) (n=6) |
| 15 - 34 | 352/193 (± 382) (n=90) | 306/193 (± 345) (n=106) | 359/185 (± 351) (n=28) | 452/339 (± 416) (n=19) |
| 35 - 59 | 369/252 (± 459) (n=333) | 353/204 (± 516) (n=216) | 425/306 (± 636) (n=130) | 406/303 (± 415) (n=86) |
| > 60 | 353/232 (± 370) (n=175) | 375/200 (± 392) (n=66) | 319/216 (± 353) (n=135) | 420/209 (± 504) (n=50) |
| **All** | 349/221 (± 416) | 328/196 (± 430) | 368/245 (± 506) | 419/289 (± 452) |
