# Supplementary material for: Characteristics and outcomes of a cohort hospitalized for pandemic and seasonal influenza in Germany based on nationwide inpatient data
Source: PLoS One. 2017 Jul 14;12(7):e0180920. doi: 10.1371/journal.pone.0180920 (PMC5510816; doi:10.1371/journal.pone.0180920)
Supplement: S4 Table — This table shows the underlying ICD codes for certain diagnoses (comorbidities) as used in S5 Table and Figs 2–4. (DOCX) [file pone.0180920.s004.docx]

| **Diagnosis** | **ICD-Code** |
| --- | --- |
| Abdominal Pain | R104 |
| Acentonemia | R824 |
| Acidosis | E872 |
| Acute Anemia | D62 |
| Acute Kidney Injury | N17 |
| Acute Respiratory Failure | J960 |
| Adipositas | E66 |
| Adynamia | R53 |
| Agranulocytosis | D70 |
| Anticoagulation | Z921 |
| Apoplex | I69, G810 |
| Apoplex | G810 |
| ARDS | J80 |
| Asthma | J45 |
| C. difficile | A047 |
| Cachexia | R64 |
| Cephalgia | R51 |
| Chest Pain | R074 |
| Chronic Anemia | D50, D64, D638 |
| Chronic Respiratory Failure | J961 |
| Conjuntivitis | H10 |
| Convulsion | G409 |
| COPD | J44 |
| Coronary Heart Disease | I25, Z951, Z955 |
| Cough | R05 |
| Dementia | F03 |
| Depression | F32 |
| Diabetes | E11 |
| Dyspnea | R060 |
| Elevated Liver Enzymes | R740 |
| Enterobacteriaceae | B962 |
| Exsiccosis | E86 |
| Febrile Seizure | R56 |
| Feeding Problems | R633 |
| Fever | R50 |
| Fungal Pneumonia | J172 |
| Gastroenteritis | A09, K52, K291 |
| GERD | K21, K449 |
| Group D Streptococci | B952 |
| HACEK Pathogens | B963 |
| Heart Failure | I50 |
| Heart Failure NYHA IV | I5014 |
| Herpes Virus | B001 |
| Hospital Aquired Pneumonia | U6900 |
| Hyperkalemia | E875 |
| Hypertension | I10, I11 |
| Hyperuricemia | E790 |
| Hypokalemia | E876 |
| Hyponatremia | E871 |
| Hypotension | I951 |
| Hypothyreoidism | E03 |
| Immunosuppression | D90 |
| Lipid Metabolism Disorder | E78 |
| Meningism | R291 |
| Myocarditis | I411 |
| Nausea | R11 |
| Nephropathy | N083, N18 |
| Nursing Related Problems | R32, R15, Z741, Z740 |
| Otitis media | H67, H66, H65 |
| Pacemaker / Device | Z950 |
| Pneumonia | J18 |
| Pregnancy | O09 |
| Pseudomonas | B965 |
| Reduced Conciousness | R400 |
| Respiratory Failure | J96 |
| S. aureus | B956 |
| SIRS / Sepsis | A419, R65 |
| Staphylococci others | B957 |
| Syncope | R55 |
| Tachyarrhythmia | I48, I47 |
| Thrombocytopenia | D69 |
| Tobacco Use | F17 |
| Tracheostoma | Z430 |
| Urinary tract infection | N390, N300 |
| Vertigo | R42 |
