# Supplementary material for: Characteristics and outcomes of a cohort hospitalized for pandemic and seasonal influenza in Germany based on nationwide inpatient data
Source: PLoS One. 2017 Jul 14;12(7):e0180920. doi: 10.1371/journal.pone.0180920 (PMC5510816; doi:10.1371/journal.pone.0180920)
Supplement: S5 Table — The absolute and relative frequencies of diagnoses in patients with seasonal and pandemic influenza are displayed. In addition, absolute and relative frequencies are shown for subpopulations of mechanically ventilated and non-ventilated patients as well as fatal and non-fatal cases. The chi-squared test was used to compare differences between seasonal and pandemic influenza (all patients) and the according p-values are stated. Empty spaces refer to counts of less than 3 and are censored in compliance with the DeStatis data protection policy. (DOCX) [file pone.0180920.s005.docx]

**Table 3. Absolute and Relative Frequencies of Comorbidities and Co-Diagnoses as retrieved from the DeStatis Database.**

|  | **Seasonal Influenza** | | | | | **Pandemic Influenza** | | | | | |  | |  |
| --- | --- | --- | --- | --- | --- | --- | --- | --- | --- | --- | --- | --- | --- | --- |
|  | **No MV** | **MV** | **Non-Fatal** | **Fatal** | **All** | | **No MV** | **MV** | **Non-Fatal** | **Fatal** | **All** | | **p for All (Seasonal vs. Pandemic)** | |
|  |  | | | |  | |  | | | | | |  | |
|  | **N (% Total)** | | | |  |  | **N (% Total)** | | | | | |  | |
| **Abdominal Pain** |  |  |  |  |  | | 141 (1.1) | 4 (0.7) |  |  | 145 (1.1) | | n.a. | |
| **Acetonemia** |  |  |  |  |  | |  |  | 181 (1.4) | 0 (0) | 181 (1.3) | | n.a. | |
| **Acidosis** | 303 (1.5) | 159 (15.4) | 340 (1.7) | 122 (21.1) | 462 (2.2) | | 182 (1.4) | 101 (16.5) | 205 (1.5) | 78 (26.6) | 283 (2.1) | | 0.3393 | |
| **Acute Anemia** | 178 (0.9) | 213 (20.7) | 257 (1.3) | 134 (23.1) | 391 (1.9) | | 120 (0.9) | 108 (17.6) | 148 (1.1) | 80 (27.3) | 228 (1.7) | | 0.1440 | |
| **Acute Renal Injury** | 308 (1.6) | 326 (31.7) | 373 (1.8) | 261 (45.1) | 634 (3) | | 173 (1.3) | 176 (28.7) | 213 (1.6) | 136 (46.4) | 349 (2.5) | | 0.0066 | |
| **Acute Respiratory Failure** | 1690 (8.5) | 715 (69.4) | 2061 (10.2) | 344 (59.4) | 2405 (11.6) | | 1036 (7.9) | 436 (71.1) | 1286 (9.6) | 186 (63.5) | 1472 (10.8) | | 0.0206 | |
| **Adipositas** | 482 (2.4) | 119 (11.6) | 549 (2.7) | 52 (9) | 601 (2.9) | | 332 (2.5) | 89 (14.5) | 386 (2.9) | 35 (11.9) | 421 (3.1) | | 0.3154 | |
| **Adynamia** | 222 (1.1) | 17 (1.7) | 227 (1.1) | 12 (2.1) | 239 (1.1) | | 140 (1.1) | 5 (0.8) | 145 (1.1) | 0 (0) | 145 (1.1) | | 0.4392 | |
| **Agranulocytosis** | 474 (2.4) | 77 (7.5) | 485 (2.4) | 66 (11.4) | 551 (2.6) | | 219 (1.7) | 46 (7.5) | 228 (1.7) | 37 (12.6) | 265 (1.9) | | < 0.0001 | |
| **Antikoagulation** | 384 (1.9) | 46 (4.5) | 405 (2) | 25 (4.3) | 430 (2.1) | | 152 (1.2) | 15 (2.4) | 158 (1.2) | 9 (3.1) | 167 (1.2) | | < 0.0001 | |
| **Apoplex** | 127 (0.6) | 19 (1.8) | 134 (0.7) | 12 (2.1) | 146 (0.7) | | 49 (0.4) | 6 (1) | 55 (0.4) | 0 (0) | 55 (0.4) | | 0.0003 | |
| **ARDS** | 239 (1.2) | 332 (32.2) | 374 (1.8) | 197 (34) | 571 (2.7) | | 175 (1.3) | 235 (38.3) | 274 (2) | 136 (46.4) | 410 (3) | | 0.1698 | |
| **Asthma** | 492 (2.5) | 12 (1.2) | 501 (2.5) | 3 (0.5) | 504 (2.4) | | 703 (5.4) | 15 (2.4) | 712 (5.3) | 6 (2) | 718 (5.2) | | < 0.0001 | |
| **Blackout** | 555 (2.8) | 8 (0.8) |  |  | 563 (2.7) | |  |  |  |  |  | | n.a. | |
| **C. difficile** | 130 (0.7) | 41 (4) | 153 (0.8) | 18 (3.1) | 171 (0.8) | | 43 (0.3) | 11 (1.8) | 51 (0.4) | 3 (1) | 54 (0.4) | | < 0.0001 | |
| **Cachexia** | 92 (0.5) | 15 (1.5) | 93 (0.5) | 14 (2.4) | 107 (0.5) | | 31 (0.2) | 8 (1.3) | 35 (0.3) | 4 (1.4) | 39 (0.3) | | 0.0013 | |
| **CAD** | 648 (3.3) | 130 (12.6) | 702 (3.5) | 76 (13.1) | 778 (3.7) | | 239 (1.8) | 35 (5.7) | 252 (1.9) | 22 (7.5) | 274 (2) | | < 0.0001 | |
| **Cephalgia** |  |  | 433 (2.1) | 0 (0) | 433 (2.1) | |  |  | 247 (1.8) | 0 (0) | 247 (1.8) | | 0.0707 | |
| **Chest Pain** |  |  |  |  |  | | 37 (0.3) | 0 (0) | 37 (0.3) | 0 (0) | 37 (0.3) | | n.a. | |
| **Chronic Anemia** | 579 (2.9) | 232 (22.5) | 692 (3.4) | 119 (20.6) | 811 (3.9) | | 338 (2.6) | 138 (22.5) | 410 (3.1) | 66 (22.5) | 476 (3.5) | | 0.0439 | |
| **Chronic Respiratory Failure** | 207 (1) | 45 (4.4) | 224 (1.1) | 28 (4.8) | 252 (1.2) | | 112 (0.9) | 20 (3.3) | 121 (0.9) | 11 (3.8) | 132 (1) | | 0.0325 | |
| **Conjunctivitis** | 355 (1.8) | 5 (0.5) | 360 (1.8) | 0 (0) | 360 (1.7) | | 120 (0.9) | 3 (0.5) |  |  | 123 (0.9) | | < 0.0001 | |
| **COPD** | 779 (3.9) | 147 (14.3) | 853 (4.2) | 73 (12.6) | 926 (4.5) | | 443 (3.4) | 89 (14.5) | 504 (3.8) | 28 (9.6) | 532 (3.9) | | 0.0109 | |
| **Cough** | 208 (1.1) | 4 (0.4) |  |  | 212 (1) | | 166 (1.3) | 6 (1) |  |  | 172 (1.3) | | 0.0395 | |
| **Dementia** |  |  | 107 (0.5) | 8 (1.4) | 115 (0.6) | |  |  | 17 (0.1) | 0 (0) | 17 (0.1) | | < 0.0001 | |
| **Depression** | 197 (1) | 34 (3.3) | 224 (1.1) | 7 (1.2) | 231 (1.1) | | 102 (0.8) | 24 (3.9) |  |  | 126 (0.9) | | 0.0885 | |
| **Diabetes** | 1033 (5.2) | 157 (15.2) | 1106 (5.5) | 84 (14.5) | 1190 (5.7) | | 413 (3.2) | 101 (16.5) | 456 (3.4) | 58 (19.8) | 514 (3.8) | | < 0.0001 | |
| **Dyspnea** | 370 (1.9) | 38 (3.7) | 392 (1.9) | 16 (2.8) | 408 (2) | | 230 (1.8) | 25 (4.1) | 246 (1.8) | 9 (3.1) | 255 (1.9) | | 0.5171 | |
| **Elevated Liver Enzymes** | 75 (0.4) | 16 (1.6) | 87 (0.4) | 4 (0.7) | 91 (0.4) | | 36 (0.3) | 4 (0.7) |  |  | 40 (0.3) | | 0.0320 | |
| **Enterobacteriacae** | 395 (2) | 113 (11) | 455 (2.2) | 53 (9.2) | 508 (2.4) | | 213 (1.6) | 52 (8.5) | 249 (1.9) | 16 (5.5) | 265 (1.9) | | 0.0019 | |
| **Epilepsy** | 137 (0.7) | 13 (1.3) | 145 (0.7) | 5 (0.9) | 150 (0.7) | | 90 (0.7) | 7 (1.1) | 92 (0.7) | 5 (1.7) | 97 (0.7) | | 0.8950 | |
| **Exsiccosis** | 3500 (17.7) | 115 (11.2) | 3533 (17.5) | 82 (14.2) | 3615 (17.4) | | 2067 (15.8) | 66 (10.8) | 2105 (15.7) | 28 (9.6) | 2133 (15.6) | | < 0.0001 | |
| **Febrile Seizure** | 1046 (5.3) | 31 (3) | 1066 (5.3) | 11 (1.9) | 1077 (5.2) | | 528 (4) | 25 (4.1) | 545 (4.1) | 8 (2.7) | 553 (4) | | < 0.0001 | |
| **Feeding Probl.** | 1393 (7) | 103 (10) | 1436 (7.1) | 60 (10.4) | 1496 (7.2) | | 647 (4.9) | 39 (6.4) | 657 (4.9) | 29 (9.9) | 686 (5) | | < 0.0001 | |
| **Fever** | 1461 (7.4) | 48 (4.7) | 1486 (7.3) | 23 (4) | 1509 (7.3) | | 861 (6.6) | 31 (5.1) | 878 (6.6) | 14 (4.8) | 892 (6.5) | | 0.0086 | |
| **Fungal Pneumonia** | 80 (0.4) | 70 (6.8) | 102 (0.5) | 48 (8.3) | 150 (0.7) | | 57 (0.4) | 49 (8) | 70 (0.5) | 36 (12.3) | 106 (0.8) | | 0.5710 | |
| **Gastroenteritis** | 1122 (5.7) | 69 (6.7) | 1164 (5.8) | 27 (4.7) | 1191 (5.7) | | 582 (4.5) | 36 (5.9) | 606 (4.5) | 12 (4.1) | 618 (4.5) | | < 0.0001 | |
| **GERD** | 177 (0.9) | 45 (4.4) | 198 (1) | 24 (4.1) | 222 (1.1) | | 75 (0.6) | 23 (3.8) | 91 (0.7) | 7 (2.4) | 98 (0.7) | | 0.0008 | |
| **Group D Streptococci** | 158 (0.8) | 92 (8.9) | 217 (1.1) | 33 (5.7) | 250 (1.2) | | 74 (0.6) | 44 (7.2) | 104 (0.8) | 14 (4.8) | 118 (0.9) | | 0.0026 | |
| **HACEK Pathogens** | 258 (1.3) | 11 (1.1) |  |  | 269 (1.3) | | 64 (0.5) | 6 (1) |  |  | 70 (0.5) | | < 0.0001 | |
| **HAP** | 146 (0.7) | 130 (12.6) | 208 (1) | 68 (11.7) | 276 (1.3) | | 76 (0.6) | 68 (11.1) | 107 (0.8) | 37 (12.6) | 144 (1.1) | | 0.0229 | |
| **Heart Failure** | 742 (3.8) | 217 (21.1) | 817 (4) | 142 (24.5) | 959 (4.6) | | 234 (1.8) | 102 (16.6) | 276 (2.1) | 60 (20.5) | 336 (2.5) | | < 0.0001 | |
| **Heart Failure NYHA IV** | 207 (1) | 117 (11.4) | 261 (1.3) | 63 (10.9) | 324 (1.6) | | 72 (0.6) | 55 (9) | 95 (0.7) | 32 (10.9) | 127 (0.9) | | < 0.0001 | |
| **Herpes Virus** | 91 (0.5) | 10 (1) | 97 (0.5) | 4 (0.7) | 101 (0.5) | | 56 (0.4) | 7 (1.1) | 58 (0.4) | 5 (1.7) | 63 (0.5) | | 0.7397 | |
| **Hyperkalemia** | 131 (0.7) | 100 (9.7) | 154 (0.8) | 77 (13.3) | 231 (1.1) | | 53 (0.4) | 47 (7.7) | 69 (0.5) | 31 (10.6) | 100 (0.7) | | 0.0004 | |
| **Hypertension** | 2381 (12) | 310 (30.1) | 2533 (12.5) | 158 (27.3) | 2691 (12.9) | | 1037 (7.9) | 148 (24.1) | 1118 (8.3) | 67 (22.9) | 1185 (8.7) | | < 0.0001 | |
| **Hyperuricemia** | 202 (1) | 23 (2.2) | 211 (1) | 14 (2.4) | 225 (1.1) | | 70 (0.5) | 8 (1.3) | 68 (0.5) | 10 (3.4) | 78 (0.6) | | < 0.0001 | |
| **Hypokalemia** | 1413 (7.1) | 380 (36.9) | 1619 (8) | 174 (30.1) | 1793 (8.6) | | 801 (6.1) | 210 (34.3) | 935 (7) | 76 (25.9) | 1011 (7.4) | | < 0.0001 | |
| **Hyponatremia** | 560 (2.8) | 88 (8.5) | 603 (3) | 45 (7.8) | 648 (3.1) | | 300 (2.3) | 59 (9.6) | 333 (2.5) | 26 (8.9) | 359 (2.6) | | 0.0079 | |
| **Hypotension** |  |  |  |  |  | |  |  | 59 (0.4) | 0 (0) | 59 (0.4) | | n.a. | |
| **Hypothyreosis** | 435 (2.2) | 70 (6.8) | 476 (2.4) | 29 (5) | 505 (2.4) | | 252 (1.9) | 36 (5.9) | 270 (2) | 18 (6.1) | 288 (2.1) | | 0.0502 | |
| **Immunosuppression** | 267 (1.4) | 53 (5.1) | 284 (1.4) | 36 (6.2) | 320 (1.5) | | 111 (0.8) | 19 (3.1) | 114 (0.9) | 16 (5.5) | 130 (0.9) | | < 0.0001 | |
| **Lipidmetabolism Disorder** | 580 (2.9) | 71 (6.9) | 620 (3.1) | 31 (5.4) | 651 (3.1) | | 248 (1.9) | 34 (5.5) | 264 (2) | 18 (6.1) | 282 (2.1) | | < 0.0001 | |
| **Meningism** |  |  | 120 (0.6) | 0 (0) | 120 (0.6) | |  |  |  |  |  | | n.a. | |
| **Myocarditis** | 68 (0.3) | 3 (0.3) | 68 (0.3) | 3 (0.5) | 71 (0.3) | | 17 (0.1) | 4 (0.7) |  |  | 21 (0.2) | | 0.0009 | |
| **Nausea** | 884 (4.5) | 30 (2.9) | 903 (4.5) | 11 (1.9) | 914 (4.4) | | 591 (4.5) | 22 (3.6) | 609 (4.5) | 4 (1.4) | 613 (4.5) | | 0.7048 | |
| **Nephropathy** | 746 (3.8) | 140 (13.6) | 788 (3.9) | 98 (16.9) | 886 (4.3) | | 272 (2.1) | 66 (10.8) | 295 (2.2) | 43 (14.7) | 338 (2.5) | | < 0.0001 | |
| **Nursing Related Problems** | 743 (3.8) | 190 (18.4) | 826 (4.1) | 107 (18.5) | 933 (4.5) | | 361 (2.8) | 110 (17.9) | 410 (3.1) | 61 (20.8) | 471 (3.4) | | < 0.0001 | |
| **Otitis media** | 851 (4.3) | 7 (0.7) |  |  | 858 (4.1) | |  |  | 278 (2.1) | 0 (0) | 278 (2) | | < 0.0001 | |
| **Pacemaker** | 153 (0.8) | 21 (2) | 156 (0.8) | 18 (3.1) | 174 (0.8) | | 53 (0.4) | 9 (1.5) | 55 (0.4) | 7 (2.4) | 62 (0.5) | | < 0.0001 | |
| **Pneumonia** | 1073 (5.4) | 237 (23) | 1191 (5.9) | 119 (20.6) | 1310 (6.3) | | 1224 (9.4) | 171 (27.9) | 1338 (10) | 57 (19.5) | 1395 (10.2) | | < 0.0001 | |
| **Pregnancy** | 286 (1.4) | 13 (1.3) | 296 (1.5) | 3 (0.5) | 299 (1.4) | | 499 (3.8) | 17 (2.8) |  |  | 516 (3.8) | | < 0.0001 | |
| **Pseudomonas** | 118 (0.6) | 60 (5.8) | 146 (0.7) | 32 (5.5) | 178 (0.9) | | 46 (0.4) | 25 (4.1) | 65 (0.5) | 6 (2) | 71 (0.5) | | 0.0003 | |
| **Reduced Conciousness** | 79 (0.4) | 19 (1.8) | 85 (0.4) | 13 (2.2) | 98 (0.5) | | 43 (0.3) | 15 (2.4) | 52 (0.4) | 6 (2) | 58 (0.4) | | 0.5222 | |
| **Respiratory failure** | 2043 (10.3) | 795 (77.2) | 2453 (12.1) | 385 (66.5) | 2838 (13.6) | | 1233 (9.4) | 457 (74.6) | 1491 (11.1) | 199 (67.9) | 1690 (12.3) | | 0.0005 | |
| **S. aureus** | 161 (0.8) | 54 (5.2) | 194 (1) | 21 (3.6) | 215 (1) | | 73 (0.6) | 22 (3.6) | 89 (0.7) | 6 (2) | 95 (0.7) | | 0.0010 | |
| **SIRS / Sepsis** | 484 (2.4) | 436 (42.3) | 639 (3.2) | 281 (48.5) | 920 (4.4) | | 275 (2.1) | 275 (44.9) | 386 (2.9) | 164 (56) | 550 (4) | | 0.0695 | |
| **Staph. Others** | 112 (0.6) | 76 (7.4) | 161 (0.8) | 27 (4.7) | 188 (0.9) | | 54 (0.4) | 38 (6.2) | 76 (0.6) | 16 (5.5) | 92 (0.7) | | 0.0191 | |
| **Tachyarrhythmia** | 780 (3.9) | 213 (20.7) | 845 (4.2) | 148 (25.6) | 993 (4.8) | | 293 (2.2) | 99 (16.2) | 324 (2.4) | 68 (23.2) | 392 (2.9) | | < 0.0001 | |
| **Thrombocytopenia** | 471 (2.4) | 201 (19.5) | 507 (2.5) | 165 (28.5) | 672 (3.2) | | 218 (1.7) | 95 (15.5) | 222 (1.7) | 91 (31.1) | 313 (2.3) | | < 0.0001 | |
| **Tobacco Use** | 246 (1.2) | 67 (6.5) | 293 (1.4) | 20 (3.5) | 313 (1.5) | | 158 (1.2) | 41 (6.7) | 188 (1.4) | 11 (3.8) | 199 (1.5) | | 0.7046 | |
| **Tracheostoma** | 123 (0.6) | 123 (11.9) | 189 (0.9) | 57 (9.8) | 246 (1.2) | | 76 (0.6) | 65 (10.6) | 113 (0.8) | 28 (9.6) | 141 (1) | | 0.1892 | |
| **Urinary Tract Infection** | 566 (2.9) | 116 (11.3) | 636 (3.1) | 46 (7.9) | 682 (3.3) | | 264 (2) | 52 (8.5) | 303 (2.3) | 13 (4.4) | 316 (2.3) | | < 0.0001 | |
| **Vertigo** | 151 (0.8) | 5 (0.5) |  |  | 156 (0.7) | | 61 (0.5) | 3 (0.5) | 64 (0.5) | 0 (0) | 64 (0.5) | | 0.0012 | |
| **Total** | 19776 (100) | 1030 (100) | 20227 (100) | 579 (100) | 20806 (100) | | 13074 (100) | 613 (100) | 13394 (100) | 293 (100) | 13687 (100) | |  | |
